# Integrating Task-Technology Fit and Community of Practice Theories to Enhance Medical Educator Skill Confidence in Generative AI: Design, Implementation, and Pilot Outcomes

**DOI:** 10.1007/s40670-025-02520-7

**Published:** 2025-10-06

**Authors:** Youngjin Cho, John L. Szarek

**Affiliations:** Department of Medical Education, Geisinger College of Health Sciences School of Medicine, 525 Pine Street, Scranton, PA 18509 USA

**Keywords:** Generative AI, Faculty development, Community of practice, Task-technology fit, Medical educators

## Abstract

Faculty need sustainable generative AI (genAI) development approaches beyond one-off workshops or resource-intensive formal programs. We piloted a community of practice (CoP) integrating task-technology fit (TTF) principles with social learning. Two volunteer facilitators delivered six sessions to medical education faculty, focusing on authentic micro-tasks with demonstrated AI-task alignment. Thirteen participants completed post-series surveys. Self-rated knowledge increased significantly (1.3 points, *p* < 0.001). Skill confidence correlated strongly with attendance (*r* = 0.78) and welcoming environment (*r* = 0.68). Most rated content as highly relevant and planned immediate use. TTF-informed CoP design rapidly increased educators’ genAI confidence through task-specific alignment and community support, offering a theory-based model for easily adoptable faculty development.

**Supplementary Information:**

The online version contains supplementary material available at 10.1007/s40670-025-02520-7.

## Background

The explosion of generative AI (genAI) onto the health-professions education scene has left many educators feeling ill-prepared to use genAI or guide responsible use by learners. Educators cite the need for protected time to learn and hands-on training in practical AI skills fitting the local context [1]. Current faculty development opportunities are often offered at one of two ends: workshops [2] or “prompt-a-thons” that engage participants in brief one-off events [3] or formalized programs that weave genAI training into multi-session curricula requiring substantial institutional support and time commitment [4].

While various other approaches show promise, most lack systematic theoretical frameworks to guide both individual skill development and community building for sustained adoption. A recent report on incorporating community of practice (CoP) into genAI addresses social learning [5] but does not address the complex interplay between individual task-performance needs and social learning mechanisms. Using the technology acceptance framework to design faculty development primarily predicts adoption intention [6, 7] rather than use and performance outcomes needed in actual educational practice [1]. AI integration in teaching requires simultaneous attention to task-technology alignment for individual performance and community-based social learning for sustained support.

Task-technology fit (TTF) theory emphasizes alignment between technology capabilities and specific user tasks as the primary driver of utilization and performance benefits [8, 9], while CoP theory demonstrates that educators learn more effectively through peer collaboration and shared practice development [10, 11]. We piloted a genAI CoP that systematically integrates TTF principles with CoP social learning to address both individual task-performance needs and community support requirements. Our TTF-informed design ensured each session demonstrated clear alignment between AI capabilities and specific educator micro-tasks (e.g., drafting questions, improving conference abstracts), while CoP principles guided our community-building approach through shared practice development, peer learning, and collective resource creation. This dual theoretical framework sought to build immediate skill confidence in medical educators through task success while fostering peer support networks.

## Activity

### Program Design

Two faculty who self-identified as regular genAI users, but with no formal training in AI-related science disciplines or faculty development, initiated the idea and volunteered to facilitate. Grounded in CoP principles (domain, community, practice) and guided by TTF considerations (ensuring genAI functions matched educator tasks), six 60-min virtual sessions were offered over an 11-week period. Our medical school employs an integrated, flipped classroom curriculum organized around clinical presentations, in which foundational sciences and clinical content are learned in the context of patient presentations. Our model emphasizes integration across disciplines and promotes engagement through application-focused, learner-centered activities. In our CoP, the domain was genAI-enabled teaching within the context of our curriculum. However, the principles discussed were generalizable regardless of curriculum structure. The community comprised Department of Medical Education (DME) faculty joining the sessions. Exemplars, prompt templates, hands-on workflows, and peer-endorsed resources were shared with the participants during the session. Each session used a concise “see one/follow along, do one, share one” flow. There was a fluid exchange of roles between participants and facilitators as learners and experts, supporting horizontal dialogue [11]. The links to the recorded session, slides, examples, prompt templates, and additional references were emailed to participants and nonparticipants in the DME after each session. Detailed learning objectives for the sessions are provided (Table 1). Following TTF principles, specific tasks such as generating clinical-vignette multiple-choice questions were selected to ensure alignment between AI capabilities and participants’ actual work requirements, maximizing both perceived usefulness and task-performance outcome. Ready-to-use prompts and beginner-friendly instructions were consistently used to create a non-threatening environment. All exercises were tested with Microsoft 365 Copilot Enterprise, which is secure and accessible to everyone in the DME. All uses of genAI within the sessions were conducted in alignment with our health system’s governance policy on AI, which provides institutional standards for responsible and secure use. These sessions were conducted between February and May 2025.

**Table 1:** Learning objectives for each session of the Generative AI Community of Practice series

Session	Title	Number of attendees	Session objectives
1	Harnessing genAI as an Educator: Let’s Level Set	24	• Describe key generative AI concepts around its use and limitations • Outline practice points for the responsible use of genAI in teaching and learning based on ethical concerns • Use a basic prompting framework to complete a chat
2	Pedagogies, Input and Output: Prompts for MCQ Writing	19	• Identify and incorporate key pedagogical principles into AI prompts for generating multiple-choice questions • Select appropriate source materials to include as context for generating vignette-style questions • Prompt the chatbot response to be exported to a Word document • Apply these skills to create at least one complete, well-structured prompt that generates pedagogically sound MCQs
3	Leveling Up: Prompt Patterns & Other Chatbots	10	• Practice prompting a chosen chatbot to provide references • Use prompt patterns that facilitate reflective thinking (e.g., “Flipped Interaction,” “Cognitive Verifier,” “Fact Check List”) to enhance generative AI interaction • Reflect on the quality of your thinking process by incorporating prompt patterns • List commercial chatbots available to the students and educators (Claude, ChatGPT, Gemini, Copilot) and describe factors to consider in choosing the chatbot to try
4	Prompting to Receive Feedback & Revise: MCQ Refinement	11	• Use a published prompt to generate a case-vignette-based MCQ • Apply structured prompting techniques to receive feedback and revise content, with specific practice using MCQ refinement as an example • Practice MCQ refinement with a prompt based on the NBME item guide and generate alternative questions • Describe how to systemically assess the quality of a genAI output using the RACCCA framework • Discuss upcoming topics of interest for the genAI Community of Practice
5	Getting the Most out of CoPilot Agents	15	• Describe what Copilot “Agents” (custom GPTs) are and when to use them • Describe criteria for packaging prompts into agents for consistency and scale • Build and configure a basic agent (naming, instruction, source integration) • Edit and share your agent within the organization • Invoke an agent mid-chat and interpret its outputs
6	Should Educators Disclose AI Use to Students?	12	• Explore when and why disclosure of AI use in teaching matters • Explain how advances in AI tools challenge trust and transparency • Practice “Line in the Sand” exercises to decide disclosure thresholds for different educator tasks • Begin forming shared norms or principles on disclosure in the educational context

This table outlines the title, number of attendees, and specific learning objectives for each of the six sessions, demonstrating a progressive skill-building approach that moves from basic AI concepts to applications tailored to the educator’s immediate tasks and local context, while incorporating ethical considerations

### Participants and Survey Collection

All DME faculty and staff were invited (~ 65 recipients) to join any or all virtual CoP sessions. The faculty of the DME includes basic science and clinician educators, the majority of whom teach in the pre-clerkship phase. The post-series effectiveness survey was administered anonymously using Qualtrics® after session 5; consequently, respondent identities or exact session attendance could not be linked to survey data.

### Measures and Analysis

Participants’ responses used a 5-point Likert scale to rate: (1) prior and current self-rated knowledge; (2) skill confidence in prompt writing, output evaluation, and workflow integration; (3) attitude change regarding initial AI concerns; (4) behavioral intentions related to genAI use; and (5) perception of an open and friendly environment. Open feedback was also included. The full survey instrument is provided in Supplementary Material 1. Paired *t*-tests evaluated pre-/post-knowledge change. Pearson’s correlations assessed relationships between survey items. Cronbach’s alpha for quantitative survey items was calculated using the Excel data analysis pack. The calculated Cronbach’s alpha was 0.85, confirming the survey items are internally consistent. The qualitative responses were limited in number (total = 18), precluding a comprehensive thematic analysis. However, illustrative comments are included herein as appropriate.

## Results and Discussion

### Attendance and Rating of Learning Atmosphere

Thirty attended at least one session, and 13 responses were received (43% response rate). The number of attendees varied between 10 and 24 per session (Table 1). The majority of respondents reported attending three or more sessions (range 2–5, median 5). Most rated the environment welcoming and non-threatening (mean 4.38 ± 0.77), and 100% agreed that sessions had the right amount of hands-on versus didactic presentation. Results of correlation studies are shown in Table 2. There was a positive correlation between the number of sessions attended and the rating of the learning environment (*r* = 0.72, *p* = 0.005).

**Table 2:** Post-series survey results to show correlations between session attendance, learning environment, and outcome measures

Survey item	Mean ± SD	Correlation w. no. of sessions attended		Correlation w. learning environment rating	
		Pearson’s *r*	*p* value	Pearson's *r*	*p* value
Prior knowledge of generative AI^#^	2.23 ± 0.60	0.45	0.12	0.12	N/A
Current knowledge^$^	3.54 ± 0.66	0.51	0.07	0.38	N/A
Knowledge gain (current–prior)	1.31 ± 0.63	0.1	0.72	0.25	N/A
Composite skill confidence	11.14 ± 1.95	**0.78**	0.0015	**0.68**	0.01
a. Writing effective prompts	3.92 ± 0.86	**0.75**	0.00344	**0.80**	0.0009
b. Evaluating/refining AI outputs	3.69 ± 0.85	**0.64**	0.018	0.58	0.039
c. Integrating generative AI into workflow	4.46 ± 0.52	**0.65**	0.015	0.27	0.36
Relevance to current work	4.53 ± 0.66	**0.65**	0.017	0.54	N/A
Intent to apply	4.77 ± 0.60	**0.72**	0.005	0.39	N/A
Likelihood of continued experimentation	4.61 ± 1.12	−0.036	N/A	−0.20	N/A
Learning environment to be welcoming and non-threatening	4.38 ± 0.77	**0.72**	0.005	N/A	N/A

This table presents descriptive statistics and correlation analyses for key survey measures (*N* = 13). Correlations examine relationships between the number of sessions attended and various outcome measures, as well as correlations between learning environment ratings and skill-confidence measures. Bolded values indicate statistically significant positive correlations (*r* >0.6 with *p* < 0.05). The difference between prior^#^ and post^$^ knowledge was statistically significantly different with a paired two-tailed t-test (*p* < 0.001). Cronbach’s alpha of quantitative survey items was 0.85

### Knowledge and Skills

Self-rated confidence in knowledge of using genAI for daily educator tasks increased significantly (mean diff = 1.31 ± 0.63, *p* < 0.001). Composite post-series skill-confidence scores (range 3–15, mean 11.15 ± 1.95) correlated strongly with the number of sessions attended (*r* = 0.78, *p* = 0.0015), suggesting that when participants could see the fit between genAI capabilities and their task completion, they became more confident, as posited in TTF. Rating of confidence in knowledge before and after session participation, or the gain measured by the difference, did not correlate with the number of sessions attended (all *r* < 0.5, *p* > 0.1, see Table 2). This finding supports TTF theory’s emphasis on task performance over declarative knowledge acquisition. The perceived “welcoming and nonthreatening” rating was strongly correlated with confidence in prompt writing and composite skills (*r* = 0.86, *p* < 0.0001; *r* = 0.68, *p* = 0.01, respectively), validating CoP theory’s emphasis on creating safe, non-judgmental environments for effective community learning [11].

### Attitude and Intent

Most respondents reported (1) the training as very/extremely relevant to their work (mean 4.53 ± 0.66), (2) that they will probably/definitely use what they learned (mean 4.77 ± 0.60), and (3) are somewhat/extremely likely to continue experimenting with AI (mean 4.61 ± 1.12). Participants’ concerns tended to decrease (46%), while some reported no initial concerns (31%) or no change (23%). The number of sessions attended is strongly correlated with the intent to use what they learned at work (*r* = 0.72, *p* = 0.005), but not with the likelihood of continued experimentation with genAI, further supporting TTF theory’s prediction that task-technology fit experiences drive specific work application intentions. However, genAI exploration appears to be influenced by additional factors beyond task-specific exposure.

### Barriers, Enablers, and Qualitative Comments

“Not having access to the necessary AI tool” was the most cited barrier (50%). This may reflect the uniqueness of our institution, which limits genAI access to a single large language model chatbot on institution-issued computers. “Regular community meetups” (70%), followed by “advanced sessions once comfortable” (54%), were the top enablers for continued confidence, validating the CoP principle on sustained community engagement for ongoing learning and support. In qualitative comments, participants valued the safe, beginner-friendly environment and hands-on practice. “Aha” moments centered on successful prompt iterations and immediate workflow applications. Suggestions for future improvement included extended practice time and ongoing community engagement.

## Discussion

Our findings demonstrate successful integration of TTF and CoP theories for genAI faculty development. A learner-friendly, peer-led community can rapidly move educators toward confident and job-relevant use of genAI. Anchoring each genAI exercise in authentic micro-tasks (TTF principle) and creating a welcoming learning atmosphere (CoP principle) were critical to building both task-specific confidence and sustained community engagement. Peer educators, without formal training in genAI, proved capable facilitators. Thus, this model can be generalized across settings without immediate access to local genAI-related expertise support or formal faculty development program. Repeated, spaced, scaffolded encounters, instead of one-off workshops, build the communities of educators likely to adopt genAI. It is critical to pre-test each prompt and workflow in settings compliant with the institution’s policy and accessible to every participant, so they can follow along seamlessly and transfer examples into their practice based on TTF principles. These theory-informed design principles ensure optimal task-technology fit while supporting community development for sustained AI integration. Study limitations include small sample size, single institution, and potential selection bias.

Our pilot study suggests that sustainability depends not just on community engagement, but on ensuring AI tools continue matching faculty’s evolving task requirements in a rapidly evolving AI context. To ensure program sustainability, we will implement three theory-informed strategies: First, we plan to offer regular AI update sessions to maintain task-technology alignment, gradually expanding to more complex educational tasks as faculty confidence grows, as indicated by the participants and TTF alignment assessments. Second, following natural CoP progression, we will transition experienced participants into peer facilitators and establish connections with formal faculty development programs or other institutional CoPs to broaden our learning community (CoP evolution). Third, we will continue to gather feedback on both individual task performance and sense of engagement to guide program evolution. We also plan to administer surveys after each session to obtain more accurate pre-/post-measurements and follow-up surveys measuring maintained use and performance gains.

In conclusion, this approach shows how medical schools can build faculty AI confidence through collaborative learning focused on actual teaching needs, requiring minimal resources while achieving meaningful outcomes. This work contributes to the faculty development literature by demonstrating the effectiveness of combining individual performance frameworks with social learning, rather than relying on single-theory approaches.

## Supplementary Information

Below is the link to the electronic supplementary material.

Supplementary Material 1 (DOCX 39.2 KB)

## Data Availability

The datasets generated during and/or analysed during the current study are available from the corresponding author on reasonable request.

## Declarations

### Generative AI Use

Generative AI tools, including Claude Sonnet 3.7, ChatGPT o3 and 4o, and Gemini 2.5 Pro, were used to brainstorm the design of survey constructs that are aligned with community of practice and task-technology fit theories and the approaches for data analysis. All outputs were thoroughly vetted, and all statistical analysis was independently conducted. We conducted the critical design and interpretation of the study, as well as the writing and refinement of the article.
