# Supplementary material for: Integrating Task-Technology Fit and Community of Practice Theories to Enhance Medical Educator Skill Confidence in Generative AI: Design, Implementation, and Pilot Outcomes
Source: Med Sci Educ. 2025 Oct 6;35(6):2705–10. doi: 10.1007/s40670-025-02520-7 (PMC12960897; doi:10.1007/s40670-025-02520-7)
Supplement: Supplementary file 1 — Supplementary Material 1 (DOCX 39.2 KB) [file 40670_2025_2520_MOESM1_ESM.docx]

Cho and Szarek, Integrating Task-Technology Fit and Community of Practice theories to enhance medical educator skill confidence in generative AI: Design, implementation, and pilot outcomes

Supplementary 1

Generative AI Community of Practice: Post-meeting Effectiveness Survey

Introduction: Thank you for participating in our Generative AI Community of Practice Get-together series. Your feedback will help us evaluate the effectiveness of this training model and improve future sessions. This survey is designed to understand your experience and is not an assessment of your performance.

Please indicate which sessions you participated in (either by attending live or watching/reviewing the recording)

1. Session 1. Level setting: Responsible AI use
2. Session 2. Pedagogies, Input and Output: Prompts for MCQ writing
3. Session 3. Level up: Prompt patterns and other chatbots
4. Session 4. Getting feedback: MCQ refinement as an example
5. Session 5: Getting most out of CoPilot Agents

Prior Knowledge 1. Rate your knowledge of using generative AI for daily educator tasks **before** participating in this community of practice series.

1. Not knowledgeable at all
2. Slightly knowledgeable
3. Moderately knowledgeable
4. Very knowledgeable
5. Extremely knowledgeable

Current Knowledge 2. Rate your current knowledge of using generative AI for daily educator tasks **after** participating in this community of practice series.

1. Not knowledgeable at all
2. Slightly knowledgeable
3. Moderately knowledgeable
4. Very knowledgeable
5. Extremely knowledgeable

Specific Skill 3. Please rate your **confidence** in the following skills **after** completing the training:     a. **Writing effective prompts for generative AI**:

1. Not at all confident
2. Slightly confident
3. Moderately confident
4. Very confident
5. Extremely confident

3. Please rate your confidence in the following skills **after** completing the training:  b. **Evaluating and refining AI-generated outputs**:

1. Not at all confident
2. Slightly confident
3. Moderately confident
4. Very confident
5. Extremely confident

3. Please rate your confidence in the following skills **after** completing the training:  c. **Integrating generative AI into your workflow:**

1. Not at all confident
2. Slightly confident
3. Moderately confident
4. Very confident
5. Extremely confident

Workplace relevance 4. How relevant is this training to your current work?

1. Not at all relevant
2. Not at all relevant
3. Moderately relevant
4. Very relevant
5. Extremely relevant

Intent to Apply 5. Will you use what you learned in this community of practice in your work?

1. Definitely not
2. Probably not
3. Might or might not
4. Probably yes
5. Definitely yes

Anticipated Barriers 6. What factors might keep you from using generative AI in your work? (Select all that apply)

1. I need additional training in generative AI
2. I will not have access to the necessary AI tools
3. I will not be provided opportunities to use what I learned
4. I will not have the time to implement what I learned
5. Lack of a policy and guideline might keep me from using generative AI
6. Concerns about AI ethics, privacy, or academic integrity might keep me from using generative AI
7. The content is not relevant to my current work
8. Other (please specify):__________________________________________________

Learning Environment 7. To what extent did you find the training environment welcoming and non-threatening for beginners?

1. Not at all welcoming
2. Slightly welcoming
3. Moderately welcoming
4. Very welcoming
5. Extremely welcoming

Learning Format 8. What is your opinion of the balance between presentation and hands-on practice?

1. Too much presentation and not enough hands-on
2. Right amount of both presentation and hands-on
3. Too much hands-on and not enough presentation

Attitude Change 9. If you had initial concerns about using generative AI, to what extent has this training changed your attitude?

1. My concerns have significantly increased
2. My concerns have significantly increased
3. No change in my concerns
4. My concerns have somewhat decreased
5. My concerns have significantly decreased
6. I did not have initial concerns

Ongoing Engagement 10. How likely are you to continue experimenting with generative AI after the training series ends?

1. Extremely unlikely
2. Somewhat unlikely
3. Neither likely nor unlikely
4. Somewhat likely
5. Extremely likely

Support Needs 11. What would help you feel confident continuing to use generative AI in your work? (Select all that apply)

1. Additional beginner-friendly training sessions
2. One-on-one coaching
3. Online discussion forum
4. Simple templates and examples to adopt
5. Online resources and guides
6. Regular community meetups
7. Advanced sessions once I'm more comfortable
8. Other (please specify): __________________________________________________

Most Valuable Aspect 12. *Please be reminded that your feedback should be anonymous and not include any identifiable information.* What aspects of the training were most helpful in making you feel comfortable with using generative AI, and why?

Improvement 13. *Please be reminded that your feedback should be anonymous and not include any identifiable information.* Please share any suggestions for improvement and/or any "aha moments" you experienced during the training.
